# A planar parabolic refractive nickel lens for high-energy X-rays

**DOI:** 10.1107/S1600577513026593

**Published:** 2013-11-27

**Authors:** Andrzej Andrejczuk, Masaru Nagamine, Yoshiharu Sakurai, Masayoshi Itou

**Affiliations:** a Faculty of Physics, University of Bialystok, Lipowa 41, 15-424 Bialystok, Poland; b Nagamine Manufacturing Co. Ltd, 1725-26 Kishinoue, Manno, Kagawa 766-0026, Japan; c Japan Synchrotron Radiation Research Institute (JASRI), SPring-8, 1-1-1 Kouto, Sayo, Hyogo 679-5198, Japan

**Keywords:** X-ray optics, compound refractive lens, nickel lens, high-energy X-rays

## Abstract

A compound refractive lens made of nickel and designed for focusing high-energy synchrotron X-rays is presented. The lens consists of 600 parabolic grooves and focuses X-rays in one plane only (planar lens). The lenses made and investigated by us earlier exhibited low transmission and irregularities in the focused beam profile. Since then, improvements in lens manufacturing technology have been made. The present lens gives an almost Gaussian profile and produces four times higher intensity at its maximum compared with the intensity of primary X-ray beams of 174 keV.

## Abstract 2

A compound refractive nickel lens focusing 174 keV X-rays to 5 µm with a gain of 4 is presented.

## 1. Introduction   

Since refraction was used for the first time to focus X-rays (Snigirev *et al.*, 1996 ▶), many different types of lenses have been manufactured and tested (see the review by Aristov & Shabelnikov, 2008 ▶). The most commonly used are parabolic lenses (Lengeler *et al.*, 1999 ▶, 2005 ▶; Schroer *et al.*, 2003 ▶). The main drawback of a parabolic refractive lens with a focal length of the order of 1 m is the small effective aperture caused by absorption in the lens material. It is most pronounced in the case of high-energy X-rays (*h*ν > 100 keV) where the theoretical effective aperture is well below 100 µm. Many efforts have been undertaken to overcome this limitation by using special designs of the optic element (*e.g.* Cederström *et al.*, 2005 ▶; Evans-Lutterodt *et al.*, 2004 ▶; Jark *et al.*, 2004 ▶; Nazmov *et al.*, 2005 ▶; Snigirev *et al.*, 2004 ▶). For example, many discrete refractive elements have been used in order to increase the effective aperture. However, the multi-element type suffers from diffractive effects as well as increased background around the main image due to higher-order maxima. Contrary to this type, the parabolic refractive lens gives a single well defined image. Moreover, its alignment and control in its experimental set-up is much easier and the performance is more stable compared with other optical elements.

High-energy X-rays are used in Compton scattering experiments, in which electron momentum density distributions in matter (mainly solid) can be investigated (Cooper *et al.*, 2004 ▶; Schülke, 2007 ▶). At beamline BL08W at SPring-8, where such experiments are routinely performed with an X-ray energy between 100 keV and 300 keV, a project began for experiments with X-rays micro-focused in the direction perpendicular to the plane of the electron orbit (one-dimensional lens). The parabolic compound refractive lens was chosen as the micro-focusing optical element because of its simple installation into existing experimental set-ups and its ability to produce focused beams with less background. Accurate one-dimensional lenses can be manufactured using the LIGA technique (Snigirev *et al.*, 2004 ▶); however, their width in the non-focusing direction does not exceed 1 mm. As will be shown later in this paper, in our set-up the horizontal size of the beam at the lens location is substantially larger, and therefore the lenses have been manufactured by pressing parabolic grooves into metal plates. First tests on lenses made of iron and nickel have been described by Andrejczuk *et al.* (2006 ▶) and Andrejczuk *et al.* (2007 ▶), respectively. The nickel lens focused X-rays at 2.7 m from the front of the lens. However, it was found that the profile of the focused beam was irregular and the X-ray transmission through the lens was substantially lower than designed.

In this paper we describe technical details and the performance of the new Ni lens which is better than previous lenses concerning the beam shape and transmission. While some basic technical data have been given by Andrejczuk *et al.* (2007 ▶), in the next chapter the manufacturing process of the lens is described in detail. In §3 the results of a test experiment with the new lens are presented and discussed. The final chapter contains a summary.

## 2. Lens manufacturing   

**Figure 1:**
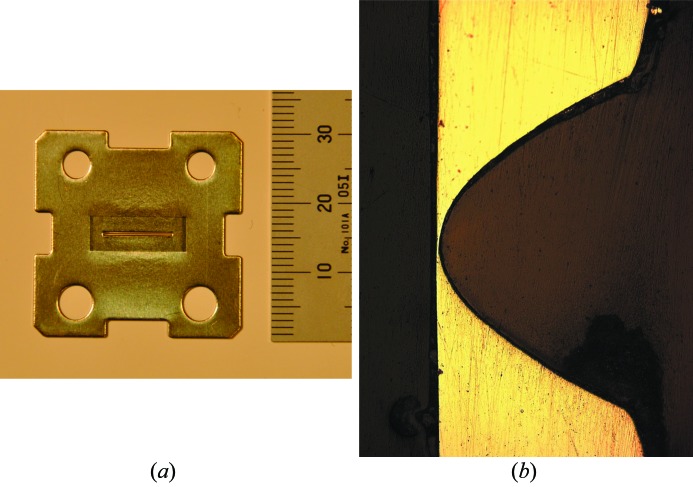
(*a*) Single nickel lens. The plate has dimensions 30 mm × 30 mm × 0.3 mm. The parabolic groove is pressed into the central part of the lens. The holes seen in the corners of the plate are used to assemble plates into a single compound lens. (*b*) Cross section of the parabolic groove obtained by cutting the lens sunk in epoxy resin. The light area in the image represents the Ni metal while the dark area is the epoxy. In the presented lens the thickness at the vertex is 2.5 µm.

The compound refractive lens consists of single Ni plates of thickness 0.3 mm in which a parabolic groove is pressed into the central part. The plate material is practically pure nickel; however, some admixture of cobalt (0.3%) and other elements (0.1% in total) exist. Before pressing, all plates are annealed at 843 K for 1 h in a vacuum. Pressing of the parabola groove is performed at the same time as punching circular holes and cutting edges of the plate to dimensions of 30 mm × 30 mm (Fig. 1*a* ▶). The punched holes are used in the assembly of the compound lens which is described later in this paragraph. The purpose of the cut-outs seen on each edge of the plates is to weld (or solder) the lens plates into one piece (bank) if necessary. The pressed groove has a length of 10 mm in order to cover the whole horizontally focused X-ray beam (Andrejczuk *et al.*, 2007 ▶). The depth of the groove is almost equal to the thickness of the plate. This can be seen in Fig. 1(*b*) ▶, where a cross section through the groove is presented. The profile of the groove is a parabola with a radius of curvature at the vertex equal to 100 µm. The width of the parabola at the open side (physical aperture) of a single lens is 480 µm.

After pressing, the lenses are annealed again. Then they are mounted (five plates at once) on the base of a polishing machine. The base has jigs that have the same parabolic shape as that pressed into the nickel plates. The space between the jig and nickel plate is filled with wax (SHIFTWAX 6607SL) at 353 K. At room temperature the wax solidifies. The rear plane of the lenses is polished with powder (solution: 200 cc water, 150 cc abrasive grain WA#8000, 30 cc coolant; Noritake Cool) for 45 min. The final phase of polishing is controlled by measuring the plate thickness with a gauge. The plates are removed from the polishing base by heating. Finally the wax is washed out using alkaline liquid in a ultrasound bath and the lens plates are dried in a drying machine.

The thickness of the single lens at the vertex is an important parameter in the case of focusing high-energy X-rays. The material at the vertex causes unnecessary additional absorption which reduces the X-ray transmission through the lens. In addition, a large number of lenses have to be used to obtain a reasonable focusing length which leads to large absorption in the material at the vertices. Therefore, our goal is to manufacture the lenses with the thickness at the vertices as small as technically possible. Inspection of ten single lenses, randomly chosen during manufacturing, showed that the mean value of the thickness at the vertices of the new lenses is 8 µm with 4 µm of standard deviation.

Andrejczuk *et al.* (2010 ▶) discussed the effects of the errors in the single lenses and their assembly on the performance of X-ray focusing. It was found that the quality of focusing is sensitive to the errors connected with the rotations of the parabola axes compared with the axis of the compound lens. Owing to the large size of the single Ni plate in the direction perpendicular to the axis of the compound lens, we believe that in our set-up the random errors in the incline of individual parabola axes to the axis of the compound lens are negligible.

**Figure 2:**
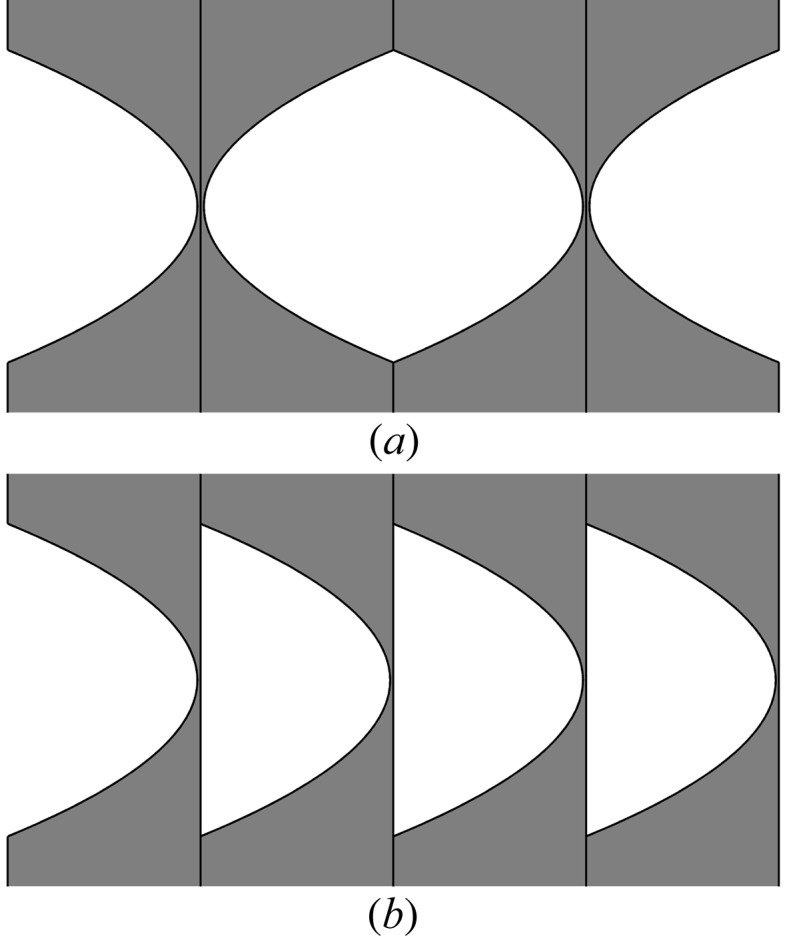
Two possible methods of the assembly of our compound lens. While the two configurations are optically equivalent, the assembly in (*a*) can average the systematic errors in the shape of the lens upon pressing. However, lenses stick by vertices and can be mechanically deformed. In the case of (*b*) the eventual systematic errors are not averaged, but the vertices are not the subject of the mechanical stress.

In the case of previous lenses, in order to minimize the influence of the systematic errors in the shape of parabolic grooves on focusing, pairs of two single lenses with opposite directions were assembled to form a block (Fig. 2*a* ▶). However, we think that the mechanical interactions between the lenses after their assembly into the compound lens were the cause of the image fragmentation reported earlier. The new lens was assembled in such a way that all lenses point in the same direction (Fig. 2*b* ▶). In the present assembly of the lens any systematic incline of the parabola axis of the pressing tool would result in an aberration, called ‘bending’ by Andrejczuk *et al.* (2010 ▶). Investigation of 30 single lenses of the previous lens, carried out by gluing the set of lenses in the epoxy resin, cutting, polishing the cut surface and measuring the section using a microscope, did not show any systematic rotation (±1 mrad) of the axes of the parabolas compared with the flat part of the lenses.

**Figure 3:**
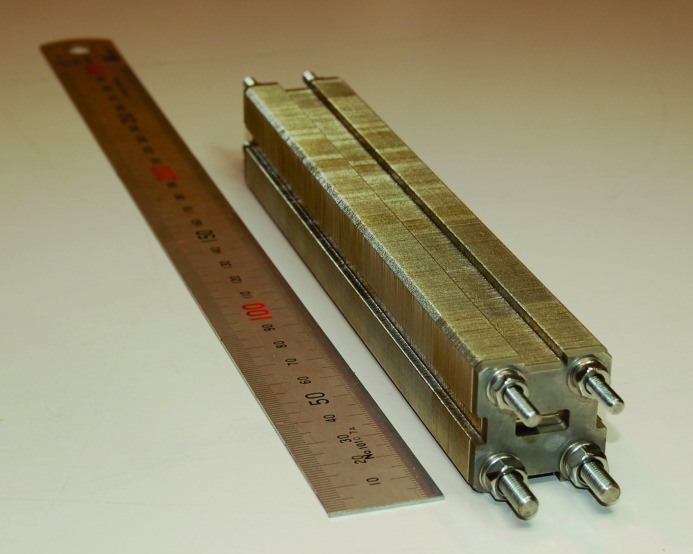
Ni compound refractive lens. The lens consist of 600 plates of 0.3 mm thickness [shown in Fig. 1(*a*) ▶]. Four assembly rods are seen in the front of the lens. The total length of the lens is 182 mm, and its total weight is 1240 g.

It was also showed by Andrejczuk *et al.* (2010 ▶) that, in order to achieve good transmission in the case of a lens with an effective aperture of 100 µm, the vertical fluctuation of the parabola vertices must be smaller than 10 µm. To accomplish this accurate alignment, each plate has circular holes in the corners made simultaneously with the pressing of the parabola, so that the positions of the holes are tightly correlated with the groove position. The holes are used to assemble the lens into one piece. There are two pairs of holes: top and bottom. The diameters of the holes are slightly different between the top and bottom ones to avoid positioning a lens in the wrong direction. Because the diameters of the rods are smaller by 10 µm than those of the corresponding holes, the accuracy of the vertical alignment of the single lenses in the compound lens can be estimated to be 5 µm. The compound lens is shown in Fig. 3 ▶. Its total length is 182 mm and its total weight is 1240 g. In the experiments the lens is mounted on a special massive flat holder (Andrejczuk *et al.*, 2006 ▶) with a bending error smaller than 1 µm in order to prevent bending of the compound lens due to gravitational forces.

## 3. Experiment and results   

**Figure 4:**
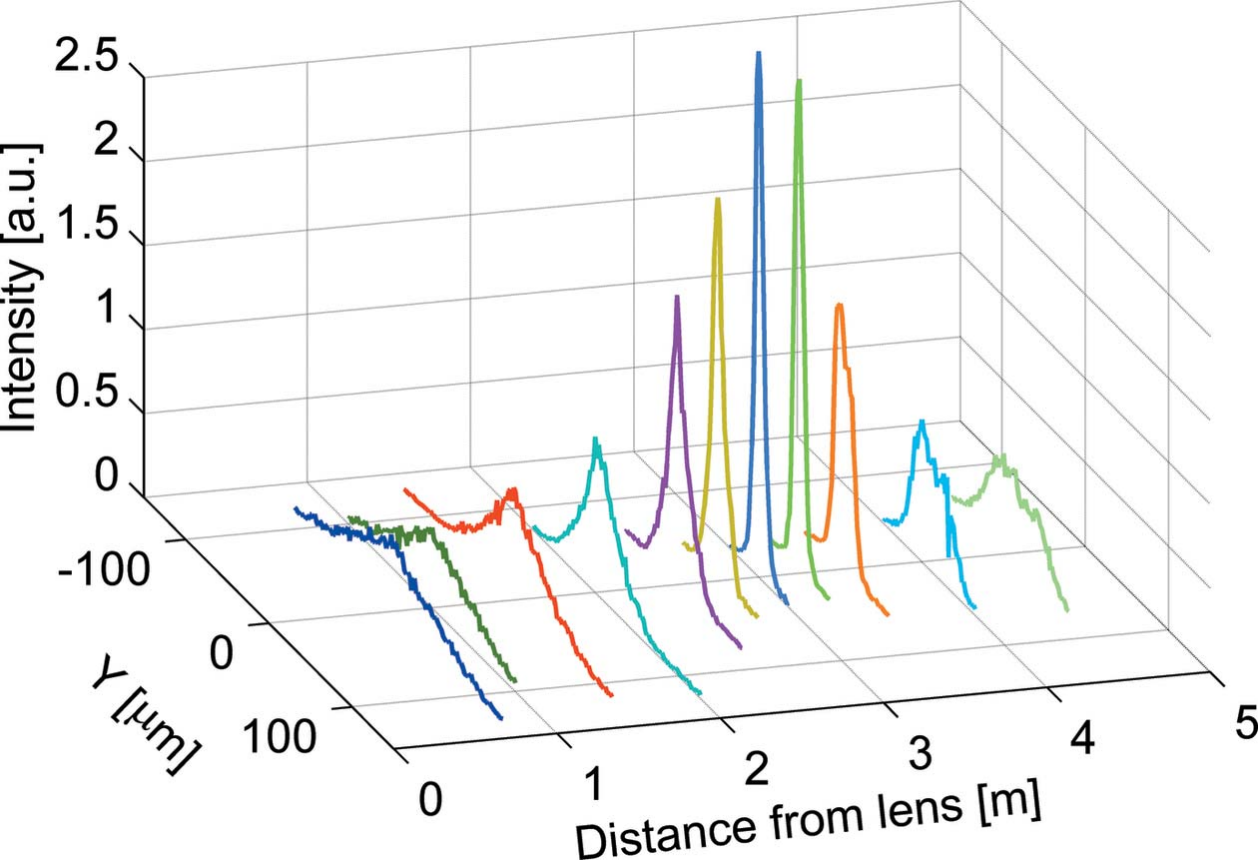
Vertical beam profile *I*(*Y*) *versus* distance from the exit of the lens. See the caption to Fig. 5 ▶.

The experimental set-up was the same as that in the previous experiment (Andrejczuk *et al.*, 2006 ▶, 2007 ▶). Radiation was emitted in the multipole wiggler. The X-ray source dimensions were estimated as 10 µm in the vertical and 200 µm in the horizontal direction. The distance from the source to the lens was 47 m. A set of filters was situated between the X-ray source and the monochromator. The experiment was performed at experimental hutch A (Sakurai, 1998 ▶; Yamaoka *et al.*, 2000 ▶). Radiation emitted from the wiggler is horizontally divergent, so the monochromator is bent to reflect as much flux as possible. The monochromator focuses the beam horizontally to a spot of ∼1 mm at the sample position, but at the entrance to the experimental hutch the horizontal size of the beam was 5 mm. During testing of the lens we reduced the beam size at the front end to 0.5 mm × 0.5 mm in order to reduce the influence of the bent monochromator on the beam shape in the vertical direction. In this experiment the monochromator reflected photons of energy 174 keV. The lens was aligned along the beam by maximizing the intensity of transmitted X-rays with the use of ion chambers. The focused beam profiles were measured with the use of a wire detector with a spatial resolution of 2 µm at several distances from the lens (Fig. 4 ▶). The results showed that the narrowest beam was approximately at 3.1 m, a greater distance by 10 cm than theoretically predicted.

**Figure 5:**
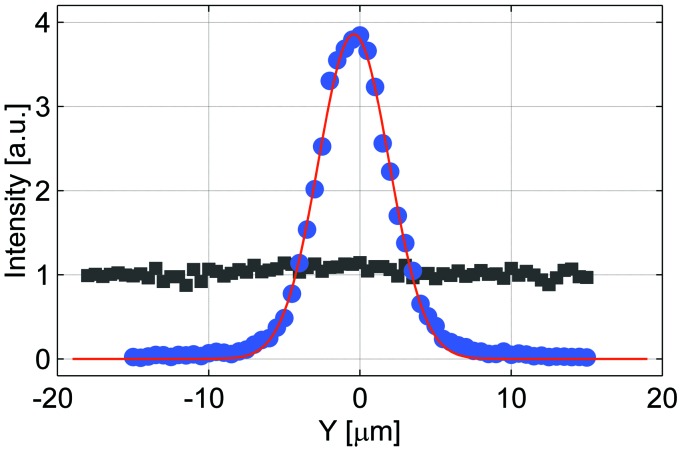
Vertical beam profile *I*(*Y*) of focused (solid circles) and unfocused (solid squares) beam at 3.1 m from the exit of the lens measured with a wire of diameter 2.5 µm. The intensity has been normalized in such a way that the intensity of the primary beam is equal to 1. The solid line represents a Gaussian fit to the focused beam. The FWHM of the fitted Gaussian is 5.5 µm.

The beam profile was measured more accurately at 3.1 m together with the primary beam intensity at the same position. The results are shown in Fig. 5 ▶. The focused beam has an almost Gaussian shape of 5.5 µm full width at half-maximum (FWHM). The maximum intensity is four times higher than that of the primary beam. When the primary beam intensity is normalized to 1 photon per 1 µm [as was done by Andrejczuk *et al.* (2010 ▶)], the integral intensity of the present focused beam is 23 photons. This is less than expected, as the calculations show that for the ideal lens with 8 µm thickness at the vertices one should obtain 40 photons.

In the present experiment the demagnification factor due to the source–lens and lens–image distances is 15. Taking into account the vertical size of the source, the image is expected to be less than 1 µm FWHM. The measured focused beam is five times as wide. It is not yet clear what is the cause of the observed broadening. Taking into account the results of the measurements of the single lenses, calculations show that the random and systematic errors in position and rotations of parabolas in the lens cannot give a broadening larger than 1 µm. Possible sources of additional broadening are optical distortions in the filters and imperfections in the lens shape or its surface roughness. Vertical distortions of the horizontally bent monochromator can be excluded because experiments with reduced bending of the monochromator did not show any changes in the shape of the focused beam apart from a reduction of the intensity.

The filters are thick (36 mm of graphite and 15 mm of aluminium). Their possible influence on the paths of X-rays has already been noted by Andrejczuk *et al.* (2007 ▶), but it was not possible to examine this since the filters were unable to be removed and a low storage-ring current operation was not possible. Investigations of the shape of the lenses made for previous Ni lens performed at an accuracy of 3 µm did not show any systematic deviation from the parabolic profile, and the roughness was better than 3 µm. The same parameters should remain for the present lens. However, it is most probable that the imperfections in the lens cause broadening of the source image and reduce the intensity (Pantell *et al.*, 2001 ▶).

The experiment has shown that the lens can already be useful. X-ray beams of 5 µm FWHM have a regular shape and can be used in scanning experiments, since no background was detected outside the focused beams. The lens is easy to align and is stable upon operation. The gain of 4 is smaller than that obtained by Snigirev and co-workers using kinoform lenses (7 at 212 keV; Snigirev *et al.*, 2004 ▶) and much smaller than the gain obtained with the one-dimensional parabolic lens made using the LIGA technique (32 at 115 keV; Nazmov *et al.*, 2007 ▶). However, it is necessary to note that such optical elements could not be used in our set-up as they cannot accept full beam of width 5 mm in the non-focusing direction. A similar optics is already available commercially (RXOPTICS, 2013 ▶). We think that the gain of our lens can be further increased by at least two times when the radius of curvature of the single lenses is reduced. A lens with a larger curvature is under development.

## 4. Summary   

The manufacturing process of parabolic compound refractive Ni lenses for high-energy X-rays at BL08W, SPring-8, has been described. The new assembly of the compound lens has been applied and a smaller thickness of the single lenses at the vertices has been achieved. The lens has produced a line focus with a height of 5 µm FWHM. A gain of 4 has already been obtained; however, some improvement is expected for the next lens.
